# Depth and Medium-Scale Spatial Processes Influence Fish Assemblage Structure of Unconsolidated Habitats in a Subtropical Marine Park

**DOI:** 10.1371/journal.pone.0096798

**Published:** 2014-05-13

**Authors:** Arthur L. Schultz, Hamish A. Malcolm, Daniel J. Bucher, Michelle Linklater, Stephen D. A. Smith

**Affiliations:** 1 National Marine Science Centre, Southern Cross University, Charlesworth Bay, Coffs Harbour, New South Wales, Australia; 2 Marine Ecosystem Research, New South Wales Department of Primary Industries, Coffs Harbour, New South Wales, Australia; 3 Marine Ecology Research Centre, School of Environment, Science and Engineering, Southern Cross University, Lismore, New South Wales, Australia; 4 Coastal and Marine Unit, Science Division, Office of Environment and Heritage, Woolongong, New South Wales, Australia; University of Windsor, Canada

## Abstract

Where biological datasets are spatially limited, abiotic surrogates have been advocated to inform objective planning for Marine Protected Areas. However, this approach assumes close correlation between abiotic and biotic patterns. The Solitary Islands Marine Park, northern NSW, Australia, currently uses a habitat classification system (HCS) to assist with planning, but this is based only on data for reefs. We used Baited Remote Underwater Videos (BRUVs) to survey fish assemblages of unconsolidated substrata at different depths, distances from shore, and across an along-shore spatial scale of 10 s of km (2 transects) to examine how well the HCS works for this dominant habitat. We used multivariate regression modelling to examine the importance of these, and other environmental factors (backscatter intensity, fine-scale bathymetric variation and rugosity), in structuring fish assemblages. There were significant differences in fish assemblages across depths, distance from shore, and over the medium spatial scale of the study: together, these factors generated the optimum model in multivariate regression. However, marginal tests suggested that backscatter intensity, which itself is a surrogate for sediment type and hardness, might also influence fish assemblages and needs further investigation. Species richness was significantly different across all factors: however, total MaxN only differed significantly between locations. This study demonstrates that the pre-existing abiotic HCS only partially represents the range of fish assemblages of unconsolidated habitats in the region.

## Introduction

To adequately represent the entire range of biota present, conservation planning for sub-tidal marine ecosystems should be based on comprehensive understanding of species, habitats, ecosystems and associated ecological processes [1]–[2]. However, in most cases, these data are not available, and hence much of the decision-making for planning and management of Marine Protected Areas (MPAs) relies on incomplete species inventories and distributions [1], [3]–[5]. New approaches that better integrate abiotic data into overall planning have consequently been advocated [1], [4], [6]–[7]. Physical and biophysical surrogates for biotic communities are now commonly used in planning of MPAs as they are more easily measured, categorised and geo-referenced [8]–[9]. Recent improvements in sonar equipment and techniques now provide high-resolution imagery of the physical properties and topography of the seabed [10]–[12]. However, the use of abiotic characteristics in planning carries the assumption that biotic patterns, which are the end-points of conservation management, consistently correlate with abiotic variables. While abiotic variables have sometimes been shown to accurately predict biotic patterns [13]–[16], this is not always the case [8]. Testing needs to be on a scale relevant to MPA planning [8], [16]–[17], and better information on biotic distributions is required for integration into existing planning and management of MPAs [4], [16].

Sub-tidal sediments are the most extensive benthic habitat worldwide [18]. However, there is a paucity of data for fish assemblages when compared with adjacent reef habitats [19]. The fact that unconsolidated habitats are critical for many commercial fisheries worldwide further emphasises the need for greater understanding of these habitats and their associated biota [20]–[21]. Often, the use of abiotic surrogates is an unavoidable strategy, due to the lack of biotic data [1], [3]–[4], [22], and the lack of fish assemblage data in unconsolidated habitats restricts objective selection of ecologically important areas for protection.

Most data on demersal fish assemblages of unconsolidated habitats have been collected by trawling [20], [23]–[24], an approach that is generally incompatible with MPA objectives [25]. In this study, we used Baited Remote Underwater Video (BRUV), which is a low-impact, remote method with increasing application to a range of sub-tidal habitats [25]–[33], to assess the effectiveness of a Habitat Classification Scheme (HCS) for representing fish assemblages of unconsolidated habitats. The current HCS for marine parks in New South Wales (NSW), Australia, is an important tool for the design and planning of multi-purpose MPAs [34] and uses a hierarchical classification, primarily based on abiotic factors: habitat type (unconsolidated sediments and hard substrata) and depth (shallow: <25 m, intermediate: 25–50 m, and deep: >50 m) [16], [34].

The Solitary Islands Marine Park (SIMP) in the Tweed-Moreton Bioregion of northern New South Wales, eastern Australia, comprises both State waters (to 3 nautical miles from shore), and the adjacent Solitary Islands Marine Reserve (SIMR) in Commonwealth waters. The SIMP is unique among NSW marine parks in that it includes several islands up to 12 km from the mainland coastline. The dominant system of currents, which includes strong influence of the East Australian Current (EAC) [35]–[36], cooler coastal counter-currents, and periodic upwelling [37], results in an overlap between temperate, tropical and subtropical endemic biota in the region [38]–[39]. The SIMP has extensive areas of both rocky reef and unconsolidated sediments from intertidal areas to depths >70 m [40]. Comprehensive surveys of fish associated with hard substrata have detected strong depth-related patterns in assemblage structure [16]: depth categories for the HCS for the SIMP (shallow: <25 m, intermediate: 25–50 m, deep: >50 m) were consequently refined to reflect these patterns. Distance from the mainland coast, which is independent of depth due to the presence of shallow fringing reef around the islands, also influences patterns of reef fish assemblages, and HCS therefore incorporates categories to represent these patterns (Inshore <1.5 km, Mid-shelf  = 1.5–6 km, Offshore >6 km).

Unconsolidated sediments dominate sub-tidal habitats in the SIMP [40], but there are currently no data to test whether the HCS adequately represents the diversity of fish communities of these habitats. However, preliminary BRUVs data, both from an examination of the effects of proximity to reef on fish assemblages [30], and from haphazard deployment across the SIMP (Malcolm, unpublished data), suggest a substantially different suite of species to those found on rocky reefs.

The focus of this research, therefore, was to examine fish assemblage structure of unconsolidated habitats to determine patterns over factors known to affect their reef counterparts (depth, distance from shore), and to determine how consistent they are over medium spatial scales (km). As data on the broad composition of unconsolidated habitats has recently been generated using swath acoustic multi-beam sidescan sonar [40], we also explored the relationship between fish assemblages and physical habitat characteristics. The specific aims were to: 1) quantify patterns of fish assemblages across different depths, distances from shore and over a medium spatial scale; and 2) determine which of these, and other environmental factors (backscatter intensity, fine-scale bathymetric range, and rugosity), are important in structuring fish assemblages of unconsolidated habitats in the SIMP using a multivariate regression model.

## Methods

**Figure 1:**
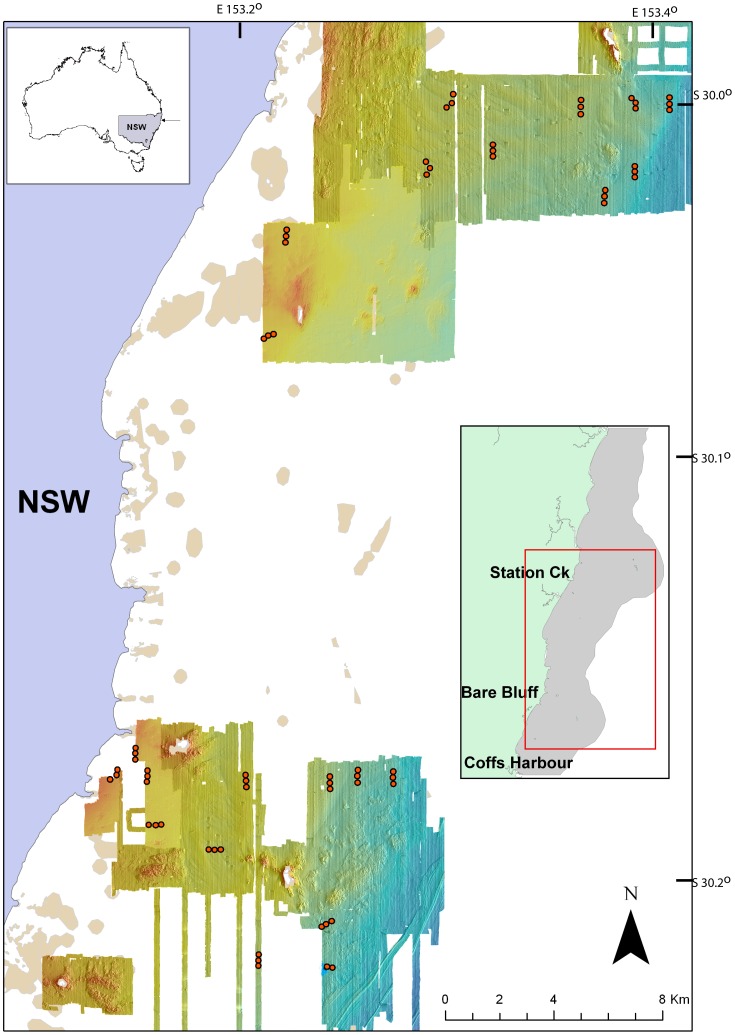
The location of the sixty-five individual BRUVs deployments in the Solitary Islands Marine Park (SIMP), New South Wales, Australia. The northern sampling site is approximately adjacent to Station Creek, and the southern sampling site is approximately adjacent to Bare Bluff. The insert box (right side of image) displays the full extent of the SIMP in grey, with the complete sampling area indicated within the red box. The second insert box (top of image) shows the study position in relation to Australia.

The SIMP covers an area of approximately 71,000 hectares, within a broad ecotone between tropical and temperate assemblages [16], [40]. The marine park is zoned for multiple use, with sections either fully protected (Sanctuary Zone), protected from commercial trawling (Habitat Protection Zone), or having no protection (General Use). Sixty-five Baited Remote Underwater Video (BRUV) deployments were completed within the SIMP during the Austral winter of 2011 (Fig. 1, Table 1). The New South Wales Marine Park Authority provided the permit for this research. As all deployments were within the SIMP boundaries, no specific permit was required for this research regarding private land access. Similarly, as all sampling was non-extractive and no collection of endangered or threatened species or other vertebrate fauna was performed, no specific permission was required in this context. BRUVs deployments were spread between two broad cross-shelf transects (‘locations’) located in the north and south of the SIMP, where the SIMP is longitudinally at its widest, and on seafloor that had previously been swath mapped using a 125 kHz GeoSwath interferometric sidescan sonar [40]. BRUV deployments were also spread across depth and distance from shore gradients that provided replication across the existing HCS categories. The design was not fully balanced due to constraints around areas that had been swath mapped: however, this did not preclude statistical modelling. Prior to the field work, coordinates for each deployment were uploaded to a handheld GPS to accurately position BRUVs in the field. There was a minimum distance of 200 m between deployments [16], [30], [41] and all deployments were made at least 400 m from the nearest reef, a distance that has been shown to be beyond the foraging range of most reef-associated fish [30].

**Table 1:** Factor groups, levels and number of BRUVs replicates sampled.

Factor	Level	Number of Replicates
Depth	10–30 m	18
	30–50 m	24
	50+m	23
Location	North	29
	South	35
Backscatter Intensity	0–50	50
	50–100	6
	100–150	8
	150+	1

### Field methods

Each BRUV drop consisted of a mini-DV video camera with a wide angle lens, within an underwater housing with flat acrylic end ports, an attachment frame, a bait pole and mesh bag with bait, and a rope and float system linking the unit to the surface [30], [41]. Approximately 1 kg of pilchard (*Sardinops neopilchardus*) was mashed into the bait bag, approximately 1.5 m from the lens of the camera, to attract fish to the viewing area in front of the camera. Each housing and bait pole was bolted to the attachment frame so that fish could be viewed in a horizontal orientation to the substratum. The field-of-view was standardised to approximately 2 m behind the bait, to minimise the effects of water clarity on measures of relative abundance [30], [41]. All files were analysed by the same person (AS) to minimise variability in estimating the field-of-view. Tapes were converted to digital format (avi) using Adobe Premiere Elements (Version 10, Adobe Systems Pty Ltd), at a resolution suitable for fish identification (720×576) [30].

### Video interrogation

Files were analysed using Eventmeasure (SeaGIS Pty Ltd, Version 3.31), and the identity and MaxN of each fish species was recorded. MaxN is the maximum number of fish of each species within the field-of-view at any one time during the 30-min recording, which removes the possibility of recounting the same fish. As counts reflect relative abundance and not density, we expect our data to be robust to variability in observing the field-of-view [41].

### Habitat factors

The placement for all BRUV deployments was determined using GIS high-24 resolution imaging software (ArcGIS v9.3, ESRI software, USA) which incorporated metrics for the factors of primary interest: depth; distance from shore; and along-shore location (as distance from latitude -29 S – hereafter termed location). Additional factors, including fine-scale bathymetric range, rugosity and backscatter were also considered. Bathymetry and backscatter data (5 m cell size) were provided by the NSW Office of Environment and Heritage (2012). Rugosity was derived from the bathymetry layer using the Benthic Terrain Modeller extension 1.2 [42]. These datasets were interrogated at a 10-m radius surrounding the BRUV point location using the Buffer tool in ArcGIS (v9.3) and Zonal Statistics tool (ArcGIS Spatial Analyst). The Zonal Statistics tool used the 10-m buffer radius to calculate cell statistics (mean, minimum, maximum, range, standard deviation and sum) for each layer within this zone. Results were exported and incorporated into the variable data for subsequent statistical analyses.

### Statistical methods

Both multivariate and univariate analyses were performed using procedures in the PRIMER 6.0 software [43], with the PERMANOVA+ add-on [44]–[45]. Distance-based linear modelling (DistLM) was conducted to model the percentage of overall variation in fish assemblage structure accounted for by each environmental factor of interest [44]–[47]. This procedure utilised a permutational approach, whereby the similarity between samples was calculated using the Bray-Curtis similarity measure, and a forward-stepping selection procedure was applied to regress the suite of environmental factors on the resultant matrix. Actual values for depth, distance from shore, location, backscatter and bathymetric range (within 10-m radius), and rugosity (averaged within 10-m radius) around each BRUV position, provided continuous abiotic data for the regression. The factor with the highest percentage influence on fish assemblage structure is selected first in this procedure, followed by the next highest ranked, in sequential order, until no further improvement to the model is made. Both adjusted R^2^ and Akaike Information Criterion (AIC) [48] selection criterion were tested in the analysis to provide a comprehensive evaluation of appropriate predictor factors to include in the model. As regression-based models are sensitive to correlation between factors [49], environmental factors with a correlation coefficient of ≥0.7 were noted, and one of the correlated pair was excluded from the analysis [49]–[50]. Such correlations were found between depth and distance from shore, and between bathymetric range and rugosity, so distance from shore and bathymetric range were discarded from the analysis, and depth and rugosity, along with location and backscatter, were retained. These variables were retained as they were considered to be the most intuitive and interpretable of the measures available.

Distance based redundancy analysis (dbRDA) biplots were generated to visually display the direction and magnitude of the relationship between habitat factors and individual fish species [47]. If DistLM indicated a significant depth effect, BRUV positions were categorised for that factor using the existing SIMP habitat classification system (shallow  = 10–30 m, intermediate  = 30–50, deep  = 50+). If location was significant, BRUV positions were categorised as north or south. If backscatter was significant it was analysed by categorising intensity range (maximum value minus minimum value) into four levels (0–50, 50–100, 100–150, and 150+) as this could be considered most analogous to sediment complexity from the values available. Likewise, if rugosity was significant it was analysed by categorising intensity range into four categories (0.00001–0.0001, 0.00011–0.001, 0.00101–0.01 and 0.01001+) as it could be considered most analogous to benthic topographic complexity. These biplots were used to visually assess the relationship between existing categories and significant factors.

Univariate permutational multivariate analysis of variance (PERMANOVA) (using Euclidean distance as the similarity measure) was performed to test for significant differences in Species Richness and Total MaxN between factors that were significant within the DistLM regression. Species Richness is calculated as the total number of different species viewed on each replicate video, and Total MaxN is calculated as the sum of MaxN for all species per replicate. Differences across bathymetry and rugosity categories were not analysed as the range of values was very low.

One-way PERMANOVA [44]–[45] was used to test for statistical differences in assemblage structure for those factors that were significant in the DistLM regression. Each test was run using 4999 permutations. Where significant results were generated, *post-hoc* pair-wise contrasts were performed to establish where differences in assemblage structure were occurring.

Multivariate assemblage structure was visually examined using non-metric multi-dimensional scaling (nMDS) ordination. Data were square-root transformed, and a dummy variable was added, prior to the generation of Bray-Curtis similarities. The resultant nMDS was used to examine differences in assemblage structure across factors that were significant in the DistLM analysis. Depth-related patterns were visually examined to see how well they fitted the current depth-based habitat classification categories [16].

## Results

### General

A total of 16 teleost and elasmobranch species, from 12 families, was recorded from BRUVs deployments. Platycephalidae was the most speciose family (3 species). Three species were numerically abundant and ubiquitous across all BRUV positions in the study – *Platycephalus caeruleopunctatus, P. longispinis* and *Aptychotrema rostrata*. Schooling *Sillago* spp. were numerically abundant in some positions, and recorded across all depths, but were highly variable between positions and between depths.

### Correlations between habitat variables and assemblage structure

**Figure 2:**
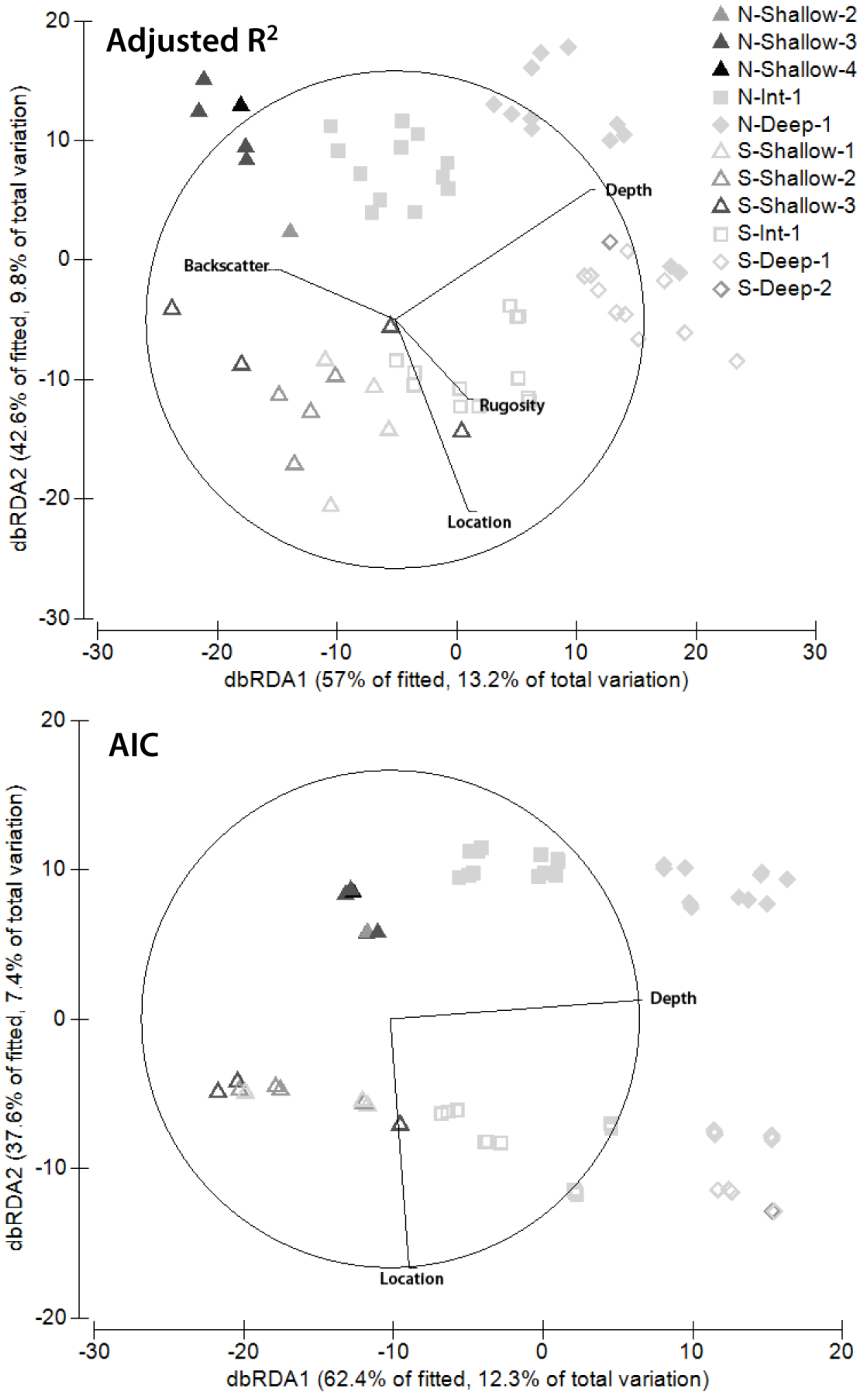
Distance-based redundancy analysis biplots representing raw Pearson correlations for habitat variables. Vectors are overlaid to represent the different environmental variables most important in each modelling approach. Length and direction of vectors indicate the strength and direction of the relationship. Numbers 1–4 within the key indicate backscatter intensity.

Marginal tests in distance-based linear modelling (DistLM) showed that, of the four environmental factors retained for analysis, three were significant in isolation (Table 2) for both the adjusted R^2^ and AIC selection criteria. However, the optimum model for adjusted R^2^ included all four factors: for AIC, only depth and location were included in the optimum model. The adjusted R^2^ model explained 23.1% of the total variation, while the AIC model explained 19.8% (Fig. 2, Table 2). The first two dbRDA axes explained 99.6% of the fitted variation for the adjusted R^2^ model, and 100% for the AIC model. In the context of hypothesis generation, the more parsimonious AIC model does not allow for further exploration of factors outside depth and location, thus it was worthwhile to further explore the adjusted R^2^ model despite it being less parsimonious. Backscatter was found to be significant in the marginal tests despite it being highly unbalanced across the study design, and thus also warranted further exploration. Raw Pearson correlations of each factor with each dbRDA axis for the adjusted R^2^ model showed depth (*ρ* = −0.90) correlated closely with the first dbRDA axis, with backscatter intensity also partially correlated (*ρ* = 0.62). The second axis was a combination of location (*ρ* = −0.85) and depth (*ρ* = −0.42). For the AIC model depth (*ρ* = −0.99) was almost perfectly correlated with the first axis, while location (*ρ* = −0.99) was similarly correlated with the second axis.

**Table 2:** Marginal and sequential test results for the distance based linear modelling (DistLM) procedure using forward selection and 4999 permutations, examining four factors of interest within the Adjusted R^2^ model, and two within the AIC model.

MARGINAL TESTS						
**Selection criterion**	**Variable**	**SS(trace)**	**Pseudo-F**	**P**	**Prop.**	****
**Adjusted R^2^**	Depth	8086.4	8.8324	**0.0002**	0.12296	
	Location	4927.5	5.1026	**0.0006**	7.49E–02	****
	Backscatter Range 10 m	3364.4	3.3966	**0.0092**	5.12E–02	****
	Rugosity	1205.7	1.1766	0.3168	1.83E–02	****
**AIC**	Depth	8086.4	8.8324	**0.001**	0.12296	****
	Location	4927.5	5.1026	**0.001**	7.49E–02	****
	Backscatter Range 10 m	3364.4	3.3966	0.011	5.12E–02	****
	Rugosity	1205.7	1.1766	0.314	1.83E–02	****

SEQUENTIAL TESTS						
**Adjusted R^2^**	**Variable**	**SS(trace)**	**Pseudo-F**	**P**	**Prop. (%)**	**Cumul. (%)**
0.10904	Depth	8086.4	8.8324	**0.0002**	0.12296	0.12296
0.17173	Location	4909.8	5.7687	**0.0002**	7.47E–02	0.19761
0.17258	Rugosity	904.3	1.0636	0.3836	1.38E–02	0.21137
0.17937	Backscatter Range 10 m	1269.2	1.5051	0.1998	1.93E–02	0.23066
**AIC**	**Variable**	**SS(trace)**	**Pseudo-F**	**P**	**Prop.**	**Cumul.**
445.24	Depth	8086.4	8.8324	**0.001**	1.23E–01	0.12296
441.45	Location	4909.8	5.7687	**0.001**	7.47E–02	0.19761

Significant values are shown in bold.

**Figure 3:**
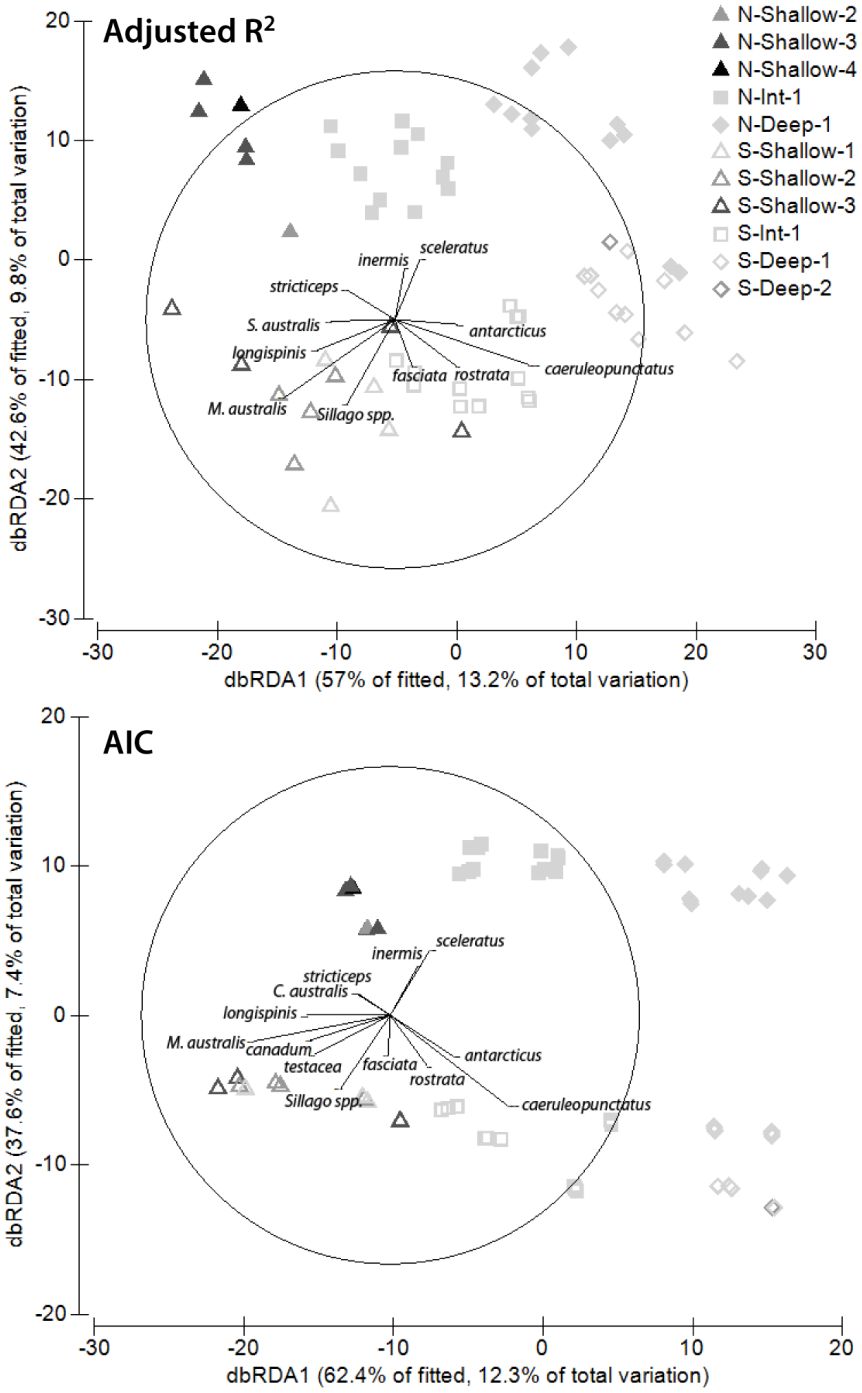
Distance-based redundancy analysis biplots representing raw Pearson correlations for fish species. This is as seen in Fig. 2, however here vectors for the 13 most influential fish species in the analysis are overlaid. Length and direction of vectors indicate the strength and direction of the relationship. Numbers 1–4 within the key indicate backscatter intensity.

By examining the raw Pearson correlations for different species, we could identify those most responsible for driving the assemblage response to each environmental factor: these are displayed as vectors in Fig. 3. The data for the adjusted R^2^ model must be interpreted somewhat cautiously, as the first dbRDA axis represents a combination of depth and backscatter, while the second axis is a combination of location and depth. Therefore, determination of the factor primarily responsible for the distribution of each species is partially confounded. Some of the species and their respective correlation values for both the adjusted R^2^ and AIC models are displayed in Table 3. Species with the four most positive and four most negative correlations with each axis are displayed, as these are the species most responsible for driving patterns in the assemblage.

**Table 3:** Family, genus, species, common name, and Pearson correlations of environmental variables with each of the four dbRDA axes, for both the adjusted R2 and AIC models.

Family	Genus	Species	Common Name	Correlation	
**RDA1**				**Adjusted R^2^**	**AIC**
Myliobatididae	*Myliobatis*	*australis*	Eagle Ray	0.44	0.55
Platycephalidae	*Platycephalus*	*longispinis*	Long-Spine Flathead	0.31	0.33
Scombridae	*Sarda*	*australis*	Australian Bonito	0.26	0.22
Urolophidae	*Trygonoptera*	*testacea*	Common Stingaree	0.19	0.30
Rachycentridae	*Rachycentron*	*canadum*	Black Kingfish	0.19	0.32
Platycephalidae	*Platycephalus*	*caeruleopunctatus*	Blue-Spotted Flathead	−0.54	−0.47
Triakidae	*Mustelus*	*antarcticus*	Gummy Shark	−0.25	−0.25
Rhinobatidae	*Aptychotrema*	*rostrata*	Eastern Shovelnose Ray	−0.25	−0.15
Aracanidae	*Anoplacapros*	*inermis*	Eastern Smooth Boxfish	−0.04	−0.11
Tetraodontidae	*Lagocephalus*	*sceleratus*	Silver Pufferfish	−0.10	−0.16
**RDA2**					
Tetraodontidae	*Lagocephalus*	*sceleratus*	Silver Pufferfish	0.24	0.26
Aracanidae	*Anoplacapros*	*inermis*	Eastern Smooth Boxfish	0.21	0.19
Pinguipedidae	*Parapercis*	*stricticeps*	White-Streaked Grubfish	0.12	0.08
Scorpaenidae	*Centropogon*	*australis*	Fortesque	0.03	0.08
Urolophidae	*Trygonoptera*	*testacea*	Common Stingaree	−0.34	−0.16
Rachycentridae	*Rachycentron*	*canadum*	Black Kingfish	−0.34	−0.10
Sillaginidae	*Sillago*	*spp.*	Whiting spp.	−0.34	−0.29
Myliobatididae	*Myliobatis*	*australis*	Eagle Ray	−0.31	−0.10
Rhinobatidae	*Aptychotrema*	*rostrata*	Eastern Shovelnose Ray	−0.19	−0.20
Rhinobatidae	*Trygonorrhina*	*fasciata*	Eastern Fiddler Ray	−0.19	−0.16
**RDA3**					
Rachycentridae	*Rachycentron*	*canadum*	Black Kingfish	0.27	
Urolophidae	*Trygonoptera*	*testacea*	Common Stingaree	0.17	
Tetraodontidae	*Lagocephalus*	*sceleratus*	Silver Pufferfish	0.16	
Aracanidae	*Anoplacapros*	*inermis*	Eastern Smooth Boxfish	0.09	
Paralichthyidae	*Pseudorhombus*	*arsius*	Large-Tooth Flounder	−0.20	
Triakidae	*Mustelus*	*antarcticus*	Gummy Shark	−0.16	
Platycephalidae	*Platycephalus*	*caeruleopunctatus*	Blue-Spotted Flathead	−0.13	
Pinguipedidae	*Parapercis*	*stricticeps*	White-Streaked Grubfish	−0.12	
**RDA4**					
Paralichthyidae	*Pseudorhombus*	*arsius*	Large-Tooth Flounder	0.39	
Myliobatididae	*Myliobatis*	*australis*	Eagle Ray	0.17	
Rhinobatidae	*Aptychotrema*	*rostrata*	Eastern Shovelnose Ray	0.10	
Pinguipedidae	*Parapercis*	*stricticeps*	White-Streaked Grubfish	0.03	
Platycephalidae	*Platycephalus*	*caeruleopunctatus*	Blue-Spotted Flathead	−0.21	
Urolophidae	*Trygonoptera*	*testacea*	Common Stingaree	−0.10	
Aracanidae	*Anoplacapros*	*inermis*	Eastern Smooth Boxfish	−0.08	
Triakidae	*Mustelus*	*antarcticus*	Gummy Shark	−0.08	

### Univariate measures

**Figure 4:**
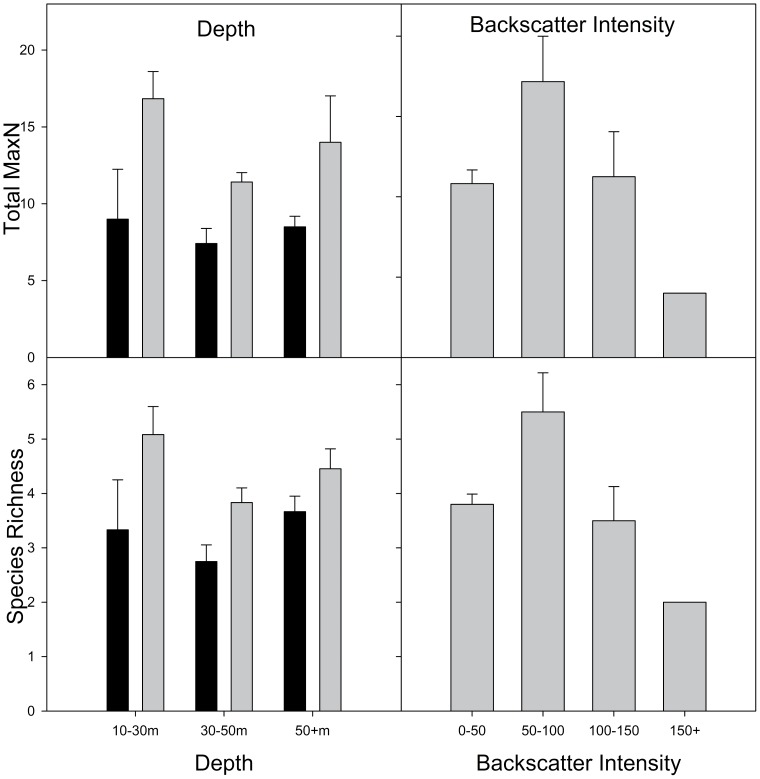
Mean species richness and total MaxN (±SE) at three depths and four different levels of backscatter intensity. The graph for depth also show differences between northern (black bars) and southern (grey bars) locations within these factors. Data from northern and southern locations has been combined for backscatter intensity due to unbalanced numbers of replicates within the four levels of intensity.

Species richness was generally higher in the south of the park (Fig. 4). The highest species richness by depth categories was for shallow deployments in the south of the park and the second highest was for deep deployments in the south. As the number of replicates was unbalanced across backscatter intensity levels (Table 1), all deployments were pooled for graphical representation. The highest species richness occurred in the 50–100 category with the lowest being in the 150+ category.

PERMANOVA revealed a significant difference between species richness for all four factors of interest (Table 4). Pairwise contrasts revealed significant differences between shallow and intermediate, and intermediate and deep categories (Table 5). The only significant contrasts for backscatter were between levels 0–50 and 50–100 (Table 5).

**Table 4:** Summary of one-way PERMANOVA results for the analysis of differences in species richness and total MaxN, and for differences in assemblage structure across the different factors.

	Source	df	SS	MS	Pseudo-F	P(perm)
**Univariate Measures**						
Species Richness	Depth	2	0.9015	0.45075	3.1429	**0.043**
	Res	62	8.8921	0.14342		
	Total	64	9.7936			
	Location	1	1.7317	1.7317	13.533	**0.001**
	Res	63	8.0618	0.12797		
	Total	64	9.7936			
	Backscatter	3	1.2872	0.42908	3.077	**0.034**
	Res	61	8.5063	0.13945		
	Total	64	9.7936			
Total MaxN	Depth	2	3.8573	1.9287	2.4472	0.092
	Res	62	48.862	0.7881		
	Total	64	52.72			
	Location	1	12.859	12.859	20.323	**0.001**
	Res	63	39.861	0.63271		
	Total	64	52.72			
	Backscatter	3	5.8483	1.9494	2.5371	0.065
	Res	61	46.871	0.76838		
	Total	64	52.72			
**Assemblage Structure**	Depth	2	8532.2	4266.1	6.8736	**0.0002**
	Res	62	38480	620.65		
	Total	64	47013			
	Location	1	5203.1	5203.1	7.8402	**0.0002**
	Res	63	41810	663.64		
	Total	64	47013			
	Backscatter	3	5905	1968.3	2.9208	**0.0016**
	Res	61	41108	673.9		
	Total	64	47013			

Significant results are shown in bold.

**Table 5:** Results of *post-hoc* pair-wise contrasts of species richness and total MaxN, and assemblage structure (PERMANOVA) for each pair of levels across the factors.

		Groups	t	P(perm)	perms
**Species Richness**	Depth	Shallow, Int.	2.0833	**0.039**	850
		Shallow, Deep	0.53341	0.598	833
		Int., Deep	2.3135	**0.029**	543
	Dist. From Sh.	Inshore, Midshelf	1.7718	0.112	477
		Inshore, Offshore	3.0799	**0.003**	378
		Midshelf, Offshore	8.43E–02	0.917	517
	Backscatter	0–50, 50–100	2.6171	**0.016**	280
		0–50, 100–150	0.7656	0.415	374
		0–50, 150+	1.4333	0.211	7
		50–100, 100–150	2.0349	0.06	222
		50–100, 150+	2.1264	0.156	5
		100–150, 150+	0.73973	0.784	6
**Assemblage Structure**	Depth	Shallow, Int.	2.377	**0.0006**	4989
		Shallow, Deep	2.8837	**0.0002**	4992
		Int., Deep	2.5972	**0.0002**	4990
	Dist. From Sh.	Inshore, Midshelf	1.9663	**0.0114**	4622
		Inshore, Offshore	3.2945	**0.0002**	4990
		Midshelf, Offshore	2.3576	**0.0016**	4987
	Backscatter	0–50, 50–100	2.5076	**0.0002**	4983
		0–50, 100–150	1.6913	**0.0258**	4983
		0–50, 150+	1.1757	0.2954	49
		50–100, 100–150	1.0779	0.346	2408
		50–100, 150+	1.414	0.1356	7
		100–150, 150+	0.55622	1	9

Significant results are shown in bold.

Total MaxN values were slightly higher in the south of the park, and showed trends similar to those seen for species richness (Fig. 4). The highest Total MaxN was recorded in the shallow southern category and the second highest in the deep southern category. As with species richness, highest Total MaxN across backscatter categories was for 50–100. Of the three factors of interest, PERMANOVA found a significant effect only for the latitude (Table 4).

### Assemblage structure

PERMANOVA revealed significant effects for all three selected factors (Table 4). Pairwise contrasts between different levels of depth were all significant. Pairwise contrasts across backscatter categories were complicated by unbalanced numbers of samples in each level, generating a large number of unique permutations between some pairs of samples, and relatively few between other pairs (Table 4), making it impossible to make statistical inferences at a level ≤0.05 for several pairs.

**Figure 5:**
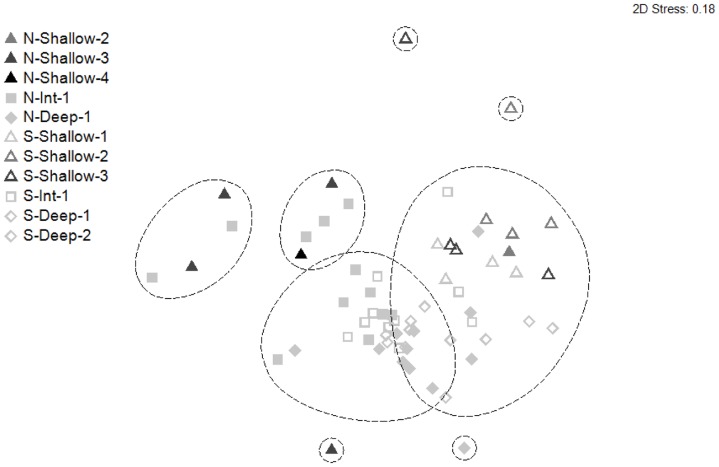
Non-metric multi-dimensional scaling (nMDS) ordination showing the relationship among fish assemblages from three depths, three distances from shore, and two locations. Data were square-root transformed prior to analysis. Lines represent 60% similarity. Numbers 1–4 within the key indicate backscatter intensity.

Some trends were evident in the nMDS ordination (Fig. 5) with the majority of shallow inshore and mid-shelf sites grouping in the top half of the plot, and the majority of the deep offshore sites in the lower half. In addition, there was some along-shore separation between assemblages, with those in northern locations tending to the left of the ordination, and those from southern locations tending to the right (Fig. 5).

## Discussion

Patterns of fish assemblage structure in unconsolidated habitats within the SIMP are clearly influenced both by depth and factors operating over medium spatial scales (i.e. the distances between our locations). Additionally, backscatter intensity, while not a primary driver, was highlighted as potentially explaining some of the differences in fish assemblages across the scales of the study.

### Assemblage patterns and correlations with environmental variables

Worldwide, fish assemblage patterns of both reef and unconsolidated habitats have often been found to be correlated with depth [16], [21], [23], [28], [32], [51]–[52] and distance from shore [26], [34], [53], and these factors have consequently been used as biophysical surrogates for conservation planning in MPAs [16], [34]. Fishes are an important ecological and socio-economic component of marine diversity in their own right [54] but can also reflect the distribution of benthic communities [16], [34]. Distinct patterns in the structure of fish assemblages of unconsolidated habitats have also been documented in previous research in coastal and shelf waters of eastern Australia [14], [19]–[20]. For example, [19] found distinct assemblages in three depth categories (20–30 m, 40–50 m and 60–70 m) on unconsolidated sediments offshore from Sydney. They also showed that: while broad patterns were evident, none of the abundant species consistently showed a preference for one depth category; species that were unique to a single depth category were found in low abundance (<3); and the numerically dominant species were found at all depths [19]. Similarly, [14] concluded that assemblage patterns were being driven mostly by differences in the abundance of common species that occurred across multiple habitats and depths. Our data show a similar pattern in that the species most responsible for differences in assemblage structure are also numerically abundant, and were recorded at all depths and within different locations in the SIMP.

The importance of depth in structuring assemblages reflects what is known from other habitats in the SIMP and elsewhere. Strong depth-related patterns have been detected in reef fishes [34], for motile invertebrates of natural [55] and artificial [56] substrata, and for habitat-forming benthos [57]. The shallow/intermediate boundary in the current HCS in the SIMP was defined at 25 m due to the disjunct patterns of reef fish and general benthic assemblages (from coral to sponge-dominated) below this depth [58].

With strong co-linearity between depth and distance from shore within our study, it is difficult to distinguish which factor primarily drives the observed patterns. Shelf position is included in the current HCS for the SIMP, as distinct cross-shelf patterns have been demonstrated for corals [59], molluscs [60] and reef fish [34]. Due to the broad morphology of the continental shelf region in the SIMP, and the mobile nature of unconsolidated habitats, this correlation between factors is not unexpected, and from a practical perspective, depth could be considered a proxy for distance from shore, or vice-versa, for management purposes.

The correlation with location in our modelling contradicts findings for reef fish communities in the SIMP [16], [34] which showed no noticeable pattern between assemblage structure over similar spatial scales, and it is currently unclear as to the reasons for this variation. While broad-scale latitudinal effects have been observed in fish communities of unconsolidated sediments [24], [26], [61]–[62], these patterns are generally evident over a much larger spatial scale than in our study (e.g. [26]). It is likely that this variation is due to other factors not identified within this study, rather than actual latitudinal effects.

Our modelling also suggests the possibility that sediment grain size, for which backscatter intensity is an appropriate surrogate, may influence fish communities of unconsolidated habitats. Broad-scale bathymetric mapping of the seafloor within the SIMP has shown that unconsolidated habitats show distinct variability in sediment type, as revealed in acoustic backscatter [40]. Some areas show a darker, ‘reef-like’ backscatter, and are likely to contain varying amounts of pebbles and cobbles [40]. These patches of denser backscatter, while extensive in some sections of the SIMP, were not the primary focus of our study. During this study, 4 species (*Pseudorhombus arsius, Parapercis stricticeps, Centropogon australis, Ratabulus diversidens*) were found only at sites with a backscatter index ≥50, despite a low number of replicates with this index or greater (15) relative to the total (65, see Table 1). Additionally, while our sampling design did not consider backscatter intensity as an *a priori* factor, the marginal tests from the DistLM analysis generated a significant value for this factor. In the context of hypothesis generation, we recommended that habitat types should be examined at a finer scale to comprehensively assess the role of backscatter intensity (as a proxy for sediment type) in the distribution of different species. With obvious and extensive differences in sediment characteristics, it is likely that, in addition to the other factors identified in this study, sediment type and habitat complexity are important factors structuring fish assemblages. Recent research in the SIMP [30] has demonstrated that the presence of reef adjacent to sedimentary habitats, even at the scale of hundreds of metres, can have a substantial effect on fish assemblage structure. It is possible that gravels may act as a ‘reef surrogate’ for some species.

When compared to reefs, unconsolidated habitats, particularly in a high energy environment, have lower topographical complexity, support fewer sessile biota, and provide limited foraging opportunities and protection from predation [63]–[65]. The low species richness observed in this study reflects this, with the species complement comprising ∼3% of the overall teleost and elasmobranch diversity recorded from the SIMP [34]. Despite this low species richness, statistical differences were evident over levels of each of the four factors we tested, and rare species were mostly restricted to single levels within each factor. In contrast, the only significant factor for total MaxN was location, with higher overall MaxN values at the southern location.

The current Habitat Classification System (HCS) for the SIMP has been developed and adjusted primarily based on fish assemblage patterns associated with hard substrata [16], [34]. This HCS has been used to systematically examine representation of habitat categories as a surrogate for biotic pattern [66]–[68], under existing and different marine park zoning scenarios [69]. In the absence of specific data, the same categories have been used for conservation planning for sediment-associated biota. There is value, both ecologically and from a planning perspective, in recognising that broad assemblage patterns in fish assemblages of unconsolidated substrata do exist in the SIMP and require consideration for planning elsewhere. That these, at least in part, align with the current habitat categories used within this MPA is opportune. However, it also indicates that some further refinement of the current HCS in the SIMP is required to improve future systematic planning. Use of quantitative and systematic planning in an MPA, based on suitable and available datasets, is more-likely to achieve MPA objectives, as well as address some of the essential criteria necessary to effectively evaluate and select areas for protection [70]–[71]. It is also more likely to provide cost-effective solutions in terms of area required and/or social impacts [71]–[72]. Biotic data are often spatially constrained, whereas physical/environmental data are often less so [66] and, therefore, integrating these can achieve better planning outcomes [73]. Without testing at the appropriate scale, however, it cannot be assumed that the patterns between fish and physical factors observed in this study will occur in other MPAs. Our study highlights not only the presence of these relationships, but also the fact that there is considerable overlap in the factors driving differences between different habitat types (i.e. unconsolidated habitats and reefs). Importantly, this study has identified additional factors, and in particular sediment morphology, which require further testing with a view to potential incorporation into the planning process.

## Conclusions

The availability of high resolution benthic mapping has allowed us to examine the influence of a range of factors, which would otherwise be difficult and expensive to assess, on the poorly described fish assemblage of unconsolidated habitats. The patterns evident within the study endorse the relevance of depth and distance from shore as categories currently used as biophysical surrogates to represent discrete assemblages in marine parks in NSW. However, the study also indicates that the inclusion of habitat type, based on backscatter and possibly other remotely-sensed metrics, will lead to better representation of assemblages of unconsolidated habitats.

## References

[1] Ward TJ, Vanderklift MA, Nicholls AO, Kenchington RA (1999) Selecting marine reserves using habitats and species assemblages as surrogates for biological diversity. Ecol Appl 9: 691–698.

[2] Curley BG, Kingsford MJ, Gillanders BM (2002) Spatial and habitat-related patterns of temperate reef fish assemblages: implications for the design of Marine Protected Areas. Mar Freshw Res 53: 1197–1210.

[3] Gladstone W (2002) The potential value of indicator groups in the selection of marine reserves. Biol Conserv 104: 211–220.

[4] Smith SDA (2005) Rapid assessment of invertebrate biodiversity on rocky shores: where there's a whelk there's a way. Biodivers Conserv 14: 3565–3576.

[5] Gladstone W (2007) Requirements for marine protected areas to conserve the biodiversity of rocky reef fishes. Aquat Conserv 17: 71–80.

[6] Stevens T (2002) Rigour and representativeness in Marine Protected Area design. Coast Manage 30: 237–248.

[7] Roberts CM, Andelman S, Branch G, Bustamante RH, Castilla JC, et al (2003) Application of ecological criteria in selecting marine reserves and developing reserve networks. Ecol Appl 13(1) Supp: S215–S228.

[8] Stevens T, Connolly RM (2004) Testing the utility of abiotic surrogates for marine habitat mapping at scales relevant to management. Biol Conserv 119: 351–362.

[9] Dalleau M, Andrefouet S, Wabnitz CCC, Payri C, Wantiez L, et al (2010) Use of Habitats as Surrogates of Biodiversity for Efficient Coral Reef Conservation Planning in Pacific Ocean Islands. Conserv Biol 24(2): 541–552. 20105207 10.1111/j.1523-1739.2009.01394.x

[10] Rattray A, Lerodiaconou D, Laurenson L, Burq S, Reston M (2009) Hydro-acoustic remote sensing of benthic biological communities on the shallow South East Australian continental shelf. Est Coast Shelf Sci 84: 237–245.

[11] Moore CH, Harvey ES, Van Niel KP (2009) Spatial prediction of demersal fish distributions: enhancing our understanding of species-environment relationships. ICES J. Mar. Sci 66: 2068–2075.

[12] Brown CJ, Smith SJ, Lawton P, Anderson JT (2011) Benthic habitat mapping: A review of progress towards improved understanding of the spatial ecology of the seafloor using acoustic techniques. Est Coast Shelf Sci 92: 502–520.

[13] Long BG, Bode L, Mason L, Pitcher CR (1997) Seabed current stress predicts the distribution and abundance of epibenthos in Torres Strait. Report to Australian Fisheries Management Authority, CSIRO, Australia.

[14] Williams A, Bax NJ (2001) Delineating fish-habitat associations for spatially based management: an example from the south-eastern Australian continental shelf. Mar Freshw Res 52: 513–536.

[15] Zacharias MA, Roff JC (2001) Explanations of patterns of intertidal diversity at regional scales. J Biogeogr 28: 471–483.

[16] Malcolm HA, Jordan A, Smith SDA (2011a) Testing a depth based Habitat Classification System against reef fish assemblage patterns in a subtropical marine park. Aquat Conserv 21: 173–185.

[17] Roff JC, Evans SMJ (2002) Frameworks for marine conservation – non-heirarchical approaches and distinctive habitats. Aquat Conserv 12: 635–648.

[18] Snelgrove PVR, Blackburn TH, Hutchings PA, Alongi DM, Grassle DM, et al (1997) The importance of marine sediment biodiversity in ecosystem processes. Ambio 26: 578–583.

[19] Connell SD, Lincoln-Smith MP (1999) Depth and the structure of assemblages of demersal fish: experimental trawling along a temperate coast. Est Coast Shelf Sci 48: 483–495.

[20] Gray CA, Otway NM (1994) Spatial and temporal differences in assemblages of demersal fishes on the inner continental shelf off Sydney, South-eastern Australia. Aust J Mar Freshw Res 45: 665–676.

[21] Garcia CB, Duarte LO, von Schiller D (1998) Demersal fish assemblages of the Gulf of Salamanca, Columbia (southern Caribbean Sea) Mar Ecol Prog Ser. 174: 13–25.

[22] Butler AJ, Rees T, Beesley P, Bax NJ (2010) Marine biodiversity in the Australian region. PLoS One 5(8): e11831. 20689847 10.1371/journal.pone.0011831 PMC2914019

[23] Hyndes GA, Platell ME, Potter IC, Lenanton RJC (1999) Does the composition of demersal fish assemblages in temperate coastal waters change with depth and undergo consistent seasonal change? Mar Biol 134: 335–352.

[24] Travers MJ, Potter IC, Clarke KR, Newman SJ, Hutchins JB (2010) The inshore fish faunas over soft substrates and reefs on the tropical west coast of Australia differ and change with latitude and bioregion. J Biogeogr 37: 148–169.

[25] Cappo M, Speare P, De'ath G (2004) Comparison of baited remote underwater video stations and prawn (shrimp) trawls for assessments of fish biodiversity in inter-reefal areas of the Great Barrier Reef Marine Park. J Exp Mar Biol Ecol 302(2): 123–152.

[26] Cappo M, De'ath G, Speare P (2007) Inter-reef vertebrate communities of the Great Barrier Reef Marine Park determined by baited remote underwater video stations. Mar Ecol Prog Ser 350: 209–221.

[27] Harvey ES, Cappo M, Butler J, Hall N, Kendrick GA (2007) How does the presence of bait as an attractant affect the performance of remote underwater video stations in assessments of demersal fish community structure? Mar Ecol Prog Ser 350: 245–254.

[28] Moore CH, Harvey ES, Van Neil K (2010) The application of predicted habitat models to investigate the spatial ecology of demersal fish assemblages. Mar Biol 157: 2717–2729.

[29] Owen V, Gladstone W (2010) The effectiveness of an abiotic classification of subtidal unvegetated habitat as a surrogate for the diversity of fish assemblages in a marine protected area. Program and Abstracts for the 2010 meeting of the Australian Marine Sciences Association (4–8 July 2010, Wollongong, New South Wales, Australia).

[30] Schultz AL, Malcolm HA, Bucher DJ, Smith SDA (2012) Effects of Reef Proximity on the Structure of Fish Assemblages of Unconsolidated Substrata. PloS ONE 7(11): e49437. 23189145 10.1371/journal.pone.0049437 PMC3500290

[31] Harvey ES, Butler JJ, McLean DL, Shand J (2012) Contrasting habitat use of diurnal and nocturnal fish assemblages in temperate Western Australia. J Exp Mar Biol Ecol 426–427: 78–86.

[32] Fitzpatrick BM, Harvey ES, Heyward AJ, Twiggs EJ, Colquhoun J (2012) Habitat Specialisation in Tropical Continental Shelf Demersal Fish Assemblages. PloS ONE 7(6): e39634. 22761852 10.1371/journal.pone.0039634 PMC3382469

[33] White J, Simpfendorfer CA, Tobin AJ, Heupel MR (2013) Application of baited remote underwater video surveys to quantify spatial distribution of elasmobranchs at an ecosystem scale. J Exp Mar Biol Ecol 448: 281–288.

[34] Malcolm HA, Smith SDA, Jordan A (2010a) Using patterns of reef fish assemblages to refine a Habitat Classification System for marine parks in NSW, Australia. Aquat Conserv 20: 83–92.

[35] Zann LP (2000) The eastern Australian region: a dynamic tropical/temperate biotone. Mar Pollut Bull 41: 188–203.

[36] Malcolm HA, Davies P, Jordan A, Smith SDA (2011b) Variation in sea temperature and the East Australian Current in the Solitary Islands region between 2001 to 2008. Deep Sea Res Part 2 Top Stud Oceanogr 58: 616–627.

[37] Roughan M, Middleton JH (2002) A comparison of observed upwelling mechanisms off the east coast of Australia. Cont Shelf Res 22: 2551–2572.

[38] Malcolm HA, Jordan A, Smith SDA (2010b) Biogeographical and cross-shelf patterns of reef fish in a transition zone. Mar Biodiv 40: 181–193.

[39] Smith SDA, Rule MJ, Harrison M, Dalton SJ (2008) Monitoring the sea change: preliminary assessment of the conservation value of nearshore reefs, and existing impacts, in a high-growth, coastal region of subtropical eastern Australia. Mar Poll Bull 56: 525–534. 10.1016/j.marpolbul.2007.11.016 18191421

[40] NSW Marine Park Authority (2010) Seabed habitat mapping in the Solitary Islands Marine Park and Jervis Bay Marine Park, 58 pp

[41] Malcolm HA, Gladstone W, Lindfield S, Wraith J, Lynch TP (2007) Spatial and temporal variation in reef fish assemblages of marine parks in New South Wales, Australia – baited video observations. Mar Ecol Prog Ser 350: 277–290.

[42] Wright DJ, Lundblad ER, Larkin EM, Rinehart RW, Murphy J, et al. (2005) ArcGIS Benthic Terrain Modeller, Corvallis, Oregon, Oregon State University, Davey Jones Locker Seafloor Mapping/Marine GIS Laboratory and NOAA Coastal Services Center. Available: http://www.csc.noaa.gov/products/btm/

[43] Clarke KR, Gorley RN (2006) PRIMER v6: user manual/tutorial. PRIMER-E, Plymouth UK.

[44] Anderson MJ (2001) A new method for non-parametric multivariate analysis of variance. Austral Ecol 26: 32–46.

[45] Anderson MJ, Gorley RN, Clarke KR (2008)PERMANOVA+ for PRIMER: guide to software and statistical methods. PRIMER-E, Plymouth UK

[46] McArdle B, Anderson MJ (2001) Fitting multivariate models to community data: a comment on distance-based redundancy analysis. Ecology 82(1): 290–297.

[47] Legendre P, Anderson MJ (1999) Distance-based Redundancy analysis: Testing multispecies responses in multifactorial ecological experiments. Ecol Monogr 69: 1–24.

[48] Akaike H (1973) Information theory as an extension of the maximum likelihood principle. Pp267–281In: Petrov BN, Caski F, editors. Proceedings, 2^nd^ International Symposium on Information Theory. Akademiai Kiado, Budapest.

[49] Leathwick JR, Elith J, Hastie T (2006) Comparative performance of generalized additive models and multivariate adaptive regression splines for statistical modelling of species distributions. Ecol Modell 199: 188–196.

[50] Moore CH, Van Niel K, Harvey ES (2011) The effect of landscape composition and configuration on the spatial distribution of temperate demersal fish. Ecography 34: 425–435.

[51] Friedlander AM, Parrish JD (1998) Habitat characteristics affecting fish assemblages on a Hawaiian coral reef. J Exp Mar Biol Ecol 224: 1–30.

[52] Anderson MJ, Millar RB (2004) Spatial variation and effects of habitat on temperate reef fish assemblages in northeastern New Zealand. J Exp Mar Biol Ecol 305: 191–221.

[53] Kendall MS, Christensen JD, Caldow C, Coyne M, Jeffery C, et al (2004) The influence of bottom type and shelf position on biodiversity of tropical fish inside a recently enlarged marine reserve. Aquat Conserv 14: 113–132.

[54] Shears NT, Babcock RC (2002) Marine reserves demonstrate top-down control of community structure on temperate reefs. Oecologica 132: 131–142. 10.1007/s00442-002-0920-x 28547276

[55] Smith SDA (1996) The macrofaunal community of *Ecklonia radiata* holdfasts: variation associated with sediment regime, sponge cover and depth. Aust J Ecol 21: 144–153.

[56] Rule M, Smith SDA (2007) Depth-associated patterns in the development of benthic assemblages on artificial substrata deployed on shallow, subtropical reefs. J Exp Mar Biol Ecol 345: 38–51.

[57] Mau R, Byrnes T, Wilson J, Zann L (1998) The distribution of selected continental shelf habitats and biotic communities in the Solitary Islands Marine Park. Southern Cross University Report to the NSW Marine Parks Authority. Lismore NSW

[58] Jordan A, Davies P, Ingleton T, Mesley E, Neilson J, et al.. (2010) Seabed habitat mapping of continental shelf waters of NSW. P. 209. NSW Department of Environment, Climate Change and Water.

[59] Harriott VJ, Smith SDA, Harrison PL (1994) Patterns of coral community structure of subtropical reefs in the Solitary Islands Marine Reserve, Eastern Australia. Mar Ecol Prog Ser 109: 67–76.

[60] Harrison MA, Smith SDA (2012) Cross-shelf variation in the structure of molluscan assemblages on shallow, rocky reefs in subtropical eastern Australia. Mar Biodivers 42(2): 203–216.

[61] Beentjes MP, Bull B, Hurst RJ, Bagley NW (2002) Demersal fish assemblages along the continental shelf and upper slope of the east coast of the south island, New Zealand. NZ J Mar Freshw Res 36: 197–223.

[62] Travers MJ, Potter IC, Clarke RK, Newman SJ (2012) Relationships between latitude and environmental conditions and the species richness, abundance and composition of tropical fish assemblages over soft substrata. Mar Ecol Prog Ser 446: 221–241.

[63] Robertson AI, Lenanton RCJ (1984) Fish community structure and food chain dynamics in the surf-zone of sandy beaches: the role of detached macrophyte detritus. J Exp Mar Biol Ecol 84: 265–283.

[64] Ayvazian SG, Hyndes GA (1995) Surf-zone fish assemblages in south-western Australia: do adjacent nearshore habitats and the warm Leeuwin Current influence the characteristics of the fish fauna? Mar Biol 122: 527–536.

[65] Hyndes GA, Lavery PS (2005) Does transported seagrass provide an important trophic link in unvegetated, nearshore areas? Est Coast Shelf Sci 63: 633–643.

[66] Williams A, Bax NJ (2001) Delineating fish-habitat associations for spatially-based management: an example from the south-eastern Australian continental shelf. Mar Freshw Res 52: 513–536.

[67] Stevens T, Connolly RM (2005) Local-scale mapping of benthic habitats to assess representation in a marine protected area. Mar Freshw Res 56: 111–123.

[68] Blamey LK, Branch GM (2009) Habitat diversity relative to wave action on rocky shores: implications for the selection of marine protected areas. Aquat Conserv 19: 645–657.

[69] Malcolm HA, Foulsham E, Pressey RL, Jordan A, Davies PL, et al (2012) Selecting zones in a marine park: Early systematic planning improves cost efficiency, combining habitat and biotic data improves effectiveness. Ocean Coast Manage 59: 1–12.

[70] Roberts CM, Branch G, Bustamante RH, Castilla JC Dugan J, et al (2003) Application of ecological criteria in selecting marine reserves and developing reserve networks. Ecol Appl 13(1): S215–S228.

[71] Possingham H, Ball I Andelman S (2000) Mathematical methods for identifying representative reserve networks. In: Ferson S, Bergman M, editors.Quantitative methods for conservation biology. Springer-Verlag, New York, pp 291–305

[72] Fernandes L, Day J, Lewis A, Slegers S, Kerrigan B, et al (2005) Establishing Representative No-Take Areas in the Great Barrier Reef: Large-Scale Implementation of Theory on Marine Protected Areas. Conserv Biol 19: 1733–1744.

[73] Ferrier S (2002) Mapping Spatial Pattern in Biodiversity for Regional Conservation Planning: Where to from Here? Syst Biol 51(2): 331–363. 12028736 10.1080/10635150252899806

